# An Early Predictive Scoring Model for In-Hospital Cardiac Arrest of Emergent Hemodialysis Patients

**DOI:** 10.3390/jcm10153241

**Published:** 2021-07-22

**Authors:** Shih-Hao Chen, Ya-Yun Cheng, Chih-Hao Lin

**Affiliations:** 1 Department of Emergency Medicine, National Cheng Kung University Hospital, College of Medicine, National Cheng Kung University, Tainan 70403, Taiwan; ytt1790@gmail.com; 2 Department of Environmental Health, T.H. Chan School of Public Health, Harvard University, Boston, MA 02115, USA; B507092063@tmu.edu.tw

**Keywords:** in-hospital cardiac arrest, hemodialysis, predictive scoring model, emergent hemodialysis, emergency department

## Abstract

Background: Patients undergoing hemodialysis are prone to cardiac arrests. Methods: This study aimed to develop a risk score to predict in-hospital cardiac arrest (IHCA) in emergency department (ED) patients undergoing emergency hemodialysis. Patients were included if they received urgent hemodialysis within 24 h after ED arrival. The primary outcome was IHCA within three days. Predictors included three domains: comorbidity, triage information (vital signs), and initial biochemical results. The final model was generated from data collected between 2015 and 2018 and validated using data from 2019. Results: A total of 257 patients, including 52 with IHCA, were analyzed. Statistical analysis selected significant variables with higher sensitivity cutoff, and scores were assigned based on relative beta coefficient ratio: K > 5.5 mmol/L (score 1), pH < 7.35 (score 1), oxygen saturation < 85% (score 1), and mean arterial pressure < 80 mmHg (score 2). The final scoring system had an area under the curve of 0.78 (*p* < 0.001) in the primary group and 0.75 (*p* = 0.023) in the validation group. The high-risk group (defined as sum scores ≥ 3) had an IHCA risk of 47.2% and 41.7%, while the low-risk group (sum scores < 3) had 18.3% and 7%, in the primary and validation databases, respectively. Conclusions: This predictive score model for IHCA in emergent hemodialysis patients could help healthcare providers to take necessary precautions and allocate resources.

## 1. Introduction

Hemodialysis patients are more vulnerable to cardiac diseases, arrhythmia, and sudden cardiac death [1,2]. The incidence of cardiac arrest was noted at 4.5–7/100,000 dialysis session [3,4]. and the prognosis is generally poor, [5]. with a 6-month survival rate as low as 11% [6]. Compared to patients undergoing regular hemodialysis, those who had intermittent urgent or emergent hemodialysis had a higher one-year mortality, a higher admission rate, and higher medical expenses [7]. Emergent hemodialysis is not rare in the emergency department (ED). Patients who underwent emergency hemodialysis in the ED had an increased risk of cardiac arrest [8].

Several risk factors for sudden cardiac arrest in regular hemodialysis patients have been proposed, including older age (≥55 years), [9], diabetes mellitus (DM) [3,4], coronary artery diseases (CADs), heart failure [2,4,10], poor nutrition or hypoalbuminemia, and acute inflammation [11,12,13]. The National Early Warning Score (NEWS) and the Modified Early Warning Score (MEWS) utilized vital signs, including respiratory rate, oxygen saturation, body temperature, blood pressure, heart rate, and level of consciousness, to predict clinical deterioration [14,15]. However, the NEWS and MEWS were designed for hospitalized patients and their predictive values for in-hospital cardiac arrest (IHCA) were barely acceptable [14,16]. Laboratory data have also shown predictive values for intra-dialysis cardiac arrest or out-of-hospital cardiac arrest in dialysis patients [4,17].

Emergent hemodialysis could be provided for patients who are critically ill or even undergoing resuscitation efforts. Statistical models to predict adverse outcomes, such as mortality and complications, may assist clinicians in taking necessary precautions. The aim of this study was to develop a risk score for predicting IHCA in patients undergoing emergency hemodialysis in the ED.

## 2. Methods

### 2.1. Data Source and Study Participants

We retrospectively reviewed patients using a hospital electronic database from a tertiary medical center in Taiwan between 1 January 2015 and 31 December 2019. Patients were included if they (1) received hemodialysis within 24 h of arrival at the ED and (2) were admitted to intensive care units or expired. Patients with known pregnancy, patients who were younger than 18 years, and traumatic patients were excluded from this study.

### 2.2. Primary Outcome Measure

The primary outcome was IHCA. The target group was emergent hemodialysis patients who experienced cardiac arrest within three days of hospital arrival, while the control group included those who did not.

### 2.3. Predictor Variables

The predictive variables were divided into three domains: patient characteristics, triage information (vital signs), and laboratory data. The data that were used in this study were obtained in the ED and were available before the emergency hemodialysis.

The first domain was composed of patient base characteristics, which included age, sex, and comorbidities, such as hypertension, DM, CAD, cerebral vascular accident (CVA), chronic kidney disease (CKD), and known malignancies.

The second domain was the initial triage data from the ED, which included blood pressure, body temperature, oximeter saturation in room air, sugar-level from finger prick, and Glasgow coma scale.

The third domain was the initial biochemistry data obtained from the ED, which included white blood cell count, hemoglobin, platelet, blood urea nitrogen, creatinine, estimated glomerular filtration rate, prothrombin time, activated partial thromboplastin time, sodium, potassium, magnesium, calcium, albumin, and arterial gas parameters, including pH value, base deficit (Beb), CO_2_, and O_2_. Missing data were treated as blank and were not included in the statistical analysis and regression models.

### 2.4. Prediction Model

The prediction model for IHCA was constructed using the three domains of patient information between 1 January 2015 and 31 December 2018. Significant variables were selected from each domain dependent on a significantly altered hazard ratio. We established the final model based on a backward stepwise selection of significant variables. To construct a scoring system with numerical values, the variable with the lowest beta coefficient was used to approximate the nearest integer. The beta coefficient of the other variables was divided by the lowest beta coefficient to obtain a relative ratio, and then rounded to an integer as well. This comprised the final model. The area under the curve (AUC) and hazard ratios were analyzed for the final scoring system.

### 2.5. Validation Model

The validation of the final model was performed using patient information between 1 January 2019 and 31 December 2019.

### 2.6. Statistical Analyses

We evaluated the differences in frequency distribution (percentage) of the categorical variables between IHCA using the chi-square test and the differences in continuous variables (presented as mean ± standard deviation [SD]) using the two-sample *t*-test.

To identify the independent risk factors for IHCA and assess their effects during the three domains separately, we used univariate Cox proportional hazard regressions followed by multivariate regressions to obtain the associated hazard ratios (HRs) and their 95% confidence intervals (CIs). In the multivariate analyses, we first constructed full models that included all the potential risk factors identified in the univariate analyses and then constructed the final models by applying the backward stepwise approach (exclusion set as *p* > 0.15). When the predicting scoring system with multivariate cox regression was built up in the final models, we might lose some participants who had missing data. Kaplan–Meier and log-rank test statistics were employed to evaluate the differences in the cumulative survival probability of IHCA between different risk factors.

Receiver-operating characteristic (ROC) curves were used to evaluate the association of IHCA by comparing the AUC and to establish the optimal cutoff for each risk factor with *p*-values, sensitivities, and specificities. ROC with AUC was also adopted to validate the predictive model with a multivariable combination.

Significant variables from each domain were selected using the algorithm. A scoring algorithm was developed by rounding the β coefficients from the multivariable regression final model, and the risk score was validated within the validation sample in 2019. The variable with the lowest coefficient was regarded as one. The β coefficient of the other variables was divided by the lowest coefficient to obtain the ratio and then rounded to an integer to obtain the score [18].

Forest plots were drawn using Microsoft Excel 2010 to calculate the effect size and 95% confidence interval, and the black circles represent the scores of the prediction model [19].

All statistical analyses were performed using SPSS software (Version 17.0; SPSS Inc., Chicago, IL, USA). All statistical tests were performed using a two-sided significance level of 0.05.

### 2.7. Ethical Consideration

The study protocol was reviewed and approved by the Institutional Review Board of the National Cheng Kung University Hospital, Taiwan (A-ER-109-067). The patient consent was waived for this study.

## 3. Results

**Figure 1:**
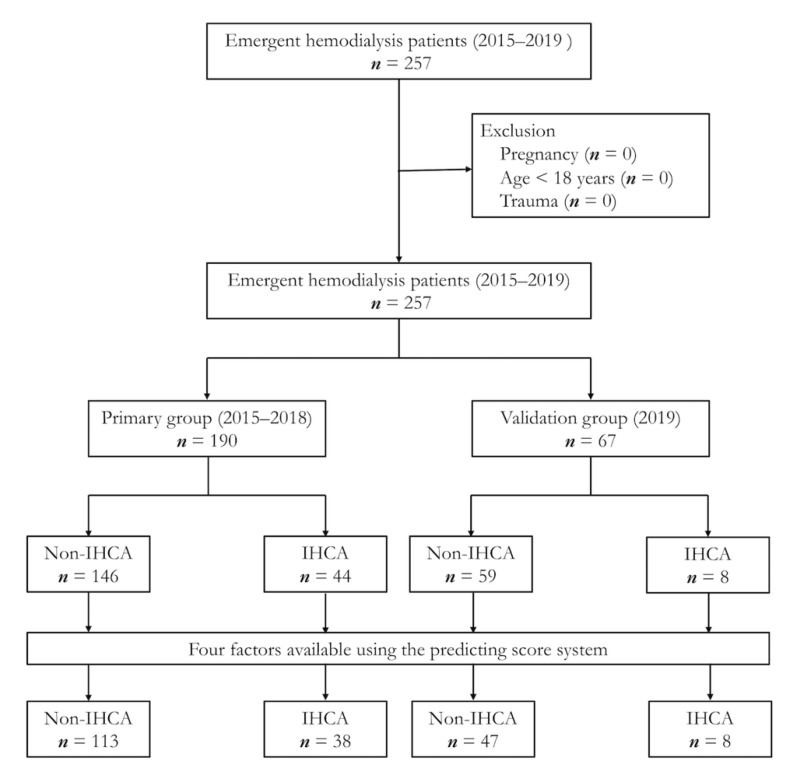
The patient flow diagram. IHCA: in-hospital cardiac arrest.

A total of 190 patients, with 44 IHCA events, between 2015 and 2018 were analyzed. A scoring system was established using the primary database. This was then validated in a 2019 database with 67 patients, of whom 8 experienced IHCA. None of the patients in the primary and validation databases met the criteria of exclusion (Figure 1).

In the primary database, the indications for emergency hemodialysis included acute pulmonary edema (53.7%), severe acidemia (46.3%), hyperkalemia (43.7%), uremic symptoms (25.8%), and others (4.7%). Some patients may have more than one indication to initiate emergency hemodialysis. The median time from ED arrival to HD onset was 7 h (range 1–24 h, interquartile range 4–14 h).

To establish the scoring system of a predictive model, we investigated variables in three domains. In the first domain, wherein patient base characteristics and comorbidities were analyzed, age and sex did not show any statistical differences between the non-IHCA and IHCA groups. Hypertension and heart failure had *p* values < 0.1 and were included in the multivariable model analysis. Hypertension had a higher incidence in the non-IHCA group than in the IHCA group (70.5% vs. 54.5%, *p* = 0.048) and stood out from the backward selection in the final model (Table 1).

**Figure 2:**
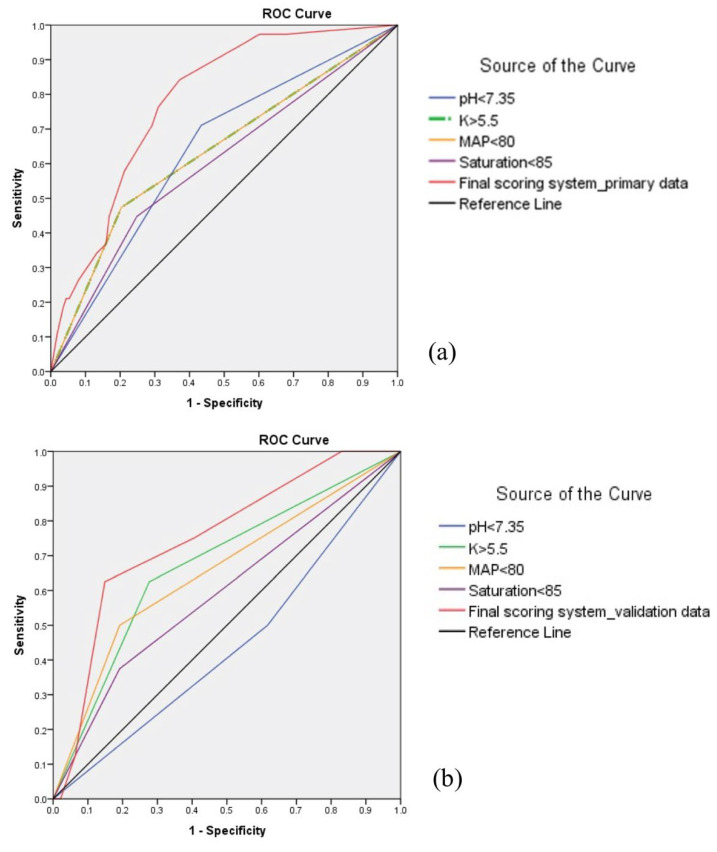
Association between predicting incidence of in-hospital cardiac arrest (IHCA) with different significant variables in each domain and combined model by using receiver-operating characteristic (ROC) curves. (**a**). Demonstration of the ROC curve to predict IHCA in the primary group collected between 2015 and 2018. The final scoring system (red line) had an area under the curve of 0.78. (**b**). Demonstration the ROC curve to predict IHCA in validation group collected in 2019. The same final scoring system (red line) had an area under the curve of 0.75.

In the second domain, wherein triage data on arrival at the ED were analyzed, blood pressure, oximeter saturation, and verbal score of the Glasgow coma scale had *p* values < 0.1 and were analyzed. Systolic blood pressure, diastolic pressure, and mean arterial pressure (MAP) were all significantly lower in the IHCA group (120 mmHg vs. 144 mmHg, 66 mmHg vs. 79 mmHg, and 84 mmHg vs. 101 mmHg, respectively) with statistical significance. Oximeter saturation was lower in the cardiac arrest group (85.8% vs. 90.4%, *p* = 0.03). Verbal score was lower in IHCA group (3.6 vs. 4.1, *p* = 0.06). Mean arterial pressure and saturation were obtained from enough statistical significance and backward selection in the final model (Table 2). We used a cutoff value with the greatest AUC of receiver ROC to determine the optimal cutoff for the continuous variable. Mean arterial pressure <80 mmHg (AUC = 0.656, *p* = 0.002), as well as oxygen saturations < 85% (AUC = 0.604, *p* = 0.048), had the greatest AUC for predicting cardiac arrest, as shown in Table S1 and Figure 2a.

In the third domain, wherein the presenting biochemical data after arriving at the ED were analyzed, white blood cell count, hemoglobin, platelet, arterial gas pH value, base deficit, and sodium, potassium, and albumin all had *p* values below 0.1 and were analyzed. Patients in the IHCA group had higher white blood cell count (14,080/μL vs. 11,920/μL, *p* = 0.03), higher hemoglobin (11.0 g/dL vs. 9.8 g/dL, *p* = 0.02), lower platelet (185,740 vs. 213,870, *p* = 0.06), higher sodium (139.0 mmol/L vs. 135.7 mmol/L, *p* = 0.005), higher potassium (5.87 mmol/L vs. 4.83 mmol/L, *p* = 0.001), lower albumin (3.0 g/dL vs. 3.8 g/dL, *p* = 0.04). Patients in the IHCA group also had more severe acidosis (pH value 7.23 vs. 7.32, *p* = 0.001) and base deficit (−10.6 vs. −6.4, *p* = 0.002) (Table 3). A pH value < 7.35 (AUC = 0.644, *p* = 0.004) and K > 5.5 mmol/L (AUC = 0.661, *p* = 0.001) had the most significant and greatest AUC during analysis and stood out in the selection, as shown in Table S1 and Figure 2a.

**Figure 3:**
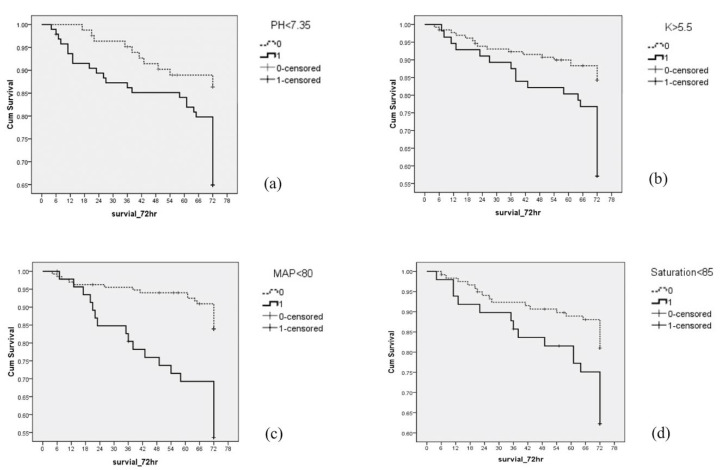
Survival analysis of the Kaplan-Meier curve and Log Rank test within the survival of IHCA from significant variables in each domain. (**a**) Survival curve differentiation of pH < 7.35 with Log Rank (Mantel-Cox) = 0.002. (**b**) Survival curve differentiation of potassium > 5.5 mmol/L with Log Rank (Mantel-Cox) < 0.001. (**c**) Survival curve differentiation of mean arterial pressure < 80 mmHg with Log Rank (Mantel-Cox) < 0.001. (**d**) Survival curve differentiation of pulse oximetry saturation < 85% with Log Rank (Mantel-Cox) = 0.01.

Significant variables that had increased hazard ratios in the three domains were analyzed to establish the final model and scoring system. Mean arterial pressure < 80 mmHg, saturation < 85%, pH < 7.35, and K > 5.5 mmol/L stood out in the final selection. Each parameter showed significant survival curve differentiations based on Kaplan-Meier analysis (Figure 3). Saturation < 85% had the lowest beta coefficient (0.515). The coefficients of the other variables were divided by 0.515, with relative ratios and were rounded to integers to obtain the hazard score. pH < 7.35, saturation < 85%, and K > 5.5 mmol/L had scores of one, respectively. Mena arterial pressure < 80 mmHg had a score of two (Table 4).

This scoring system, which consisted of four factors (i.e., pH, oxygen saturation, K, and mean arterial pressure), had a maximum total score of 5 and a minimum score of 0. The complete data of the 4 factors were available in 151 patients (including 113 non-IHCA and 38 IHCA) of the primary database and 55 patients (including 47 non-IHCA and 8 IHCA) in the validation database. This scoring system had an AUC of 0.78 (*p* < 0.001) in the primary database, as shown in Figure 2a and an AUC of 0.75 (*p* = 0.023) in the validation database, as shown in Figure 2b. The relative risk of cardiac arrest increased from 2.7% to 66.7% when the sum score was increased from 0 to 5 (Table 5). A cutoff score of ≥ 3 was defined as the high-risk group with consistent statistical significance over the primary and validation analyses. The risk of IHCA was 47.2% in the high-risk group versus 18.3% in the low-risk group in the primary analysis and was 41.7% vs. 7% in the validation database (Table 5).

The patient characteristics, including age, gender, and comorbidities, did not have significant differences between the primary and validation databases. The sensitivity analysis of major factors was performed between the primary and validation cohorts (Table S2) and between the missing-data and complete-data groups (Table S3).

## 4. Discussion

This study determined a practical score for predicting IHCA in patients undergoing emergent hemodialysis. The identified variables with cutoff values included: K > 5.5 mmol/L (score 1), pH < 7.35 (score 1), oxygen saturation on room air < 85% (score 1), and MAP < 80 mmHg (score 2). Necessary precautions should be taken for patients with a sum score of 3 or above, since these patients had a higher risk of developing IHCA.

Mean arterial pressure < 80 mmHg was determined to render patients at significant risk for cardiac arrest in the analysis. This had a better AUC value than MAP < 65 mmHg for predicting IHCA. Previous studies have also discussed optimal blood pressure targets to decrease patient mortality. Increasing MAP from 65 mmHg to a normal level was associated with improved microcirculation in hypertensive septic shock patients [20]. Mean arterial pressure < 82 mmHg was also observed to have increased mortality in patients starting continuous renal replacement therapy [21]. The optimal MAP can be further determined in the future for emergent hemodialysis patients.

Hyperkalemia increases the risk of IHCA in emergent hemodialysis patients. Hyperkalemia is a potentially life-threatening condition that impairs heart function and muscle contractility. Potassium levels, even at only moderate increases above normal, are associated with increased risk of death on critical care initiation [22]. Physiologic adaptation, heart function, medication, and comorbidity might predispose certain patients presenting with hyperkalemia to a lower or higher threshold for toxicity [23]. The process and etiology of hyperkalemia cannot be overlooked.

Acidosis with a pH value < 7.35 predicted IHCA in the analysis. Acidosis can result from muscles wasting, bone disease, hypoalbuminemia, inflammation, or progression of kidney disease [24]. This study demonstrated that only minimal acidosis may increase IHCA risk in critically ill patients. The use of sodium bicarbonate is no longer warranted in routine resuscitation practice. It increases blood pH, base deficit, serum HCO3^−^, decreased anion gap, and K, yet did not show consistency in reducing mortality [25]. Measures to correct acidosis, including emergent hemodialysis and other processes, may be further justified.

Traditional risk factors for underlying diseases, such as coronary artery disease, heart failure, or diabetes mellitus, were not associated with increased cardiac arrest in previous studies [2,4,10]. This study observed the same trend as there was no statistical difference noted for diabetes mellitus, coronary artery disease, cardiovascular accident, or heart failure. There was also no prominent correlation between the stage of chronic kidney disease and the rate of IHCA.

Some variables in the biochemistry category were statistically significant, but they were not included in the final model. The white blood cell count was elevated in the cardiac arrest group compared to that in the control group. A cutoff using the traditional definition of white count over 10,000 or below 4000 and other nearby values did not show superior AUC for prediction of IHCA. Although it did not come out in the final model, the elevated white cell count in the cardiac arrest group may indicate a more severe inflammatory or infectious state in these patients, leading to unpleasant outcomes.

Low albumin levels were significantly correlated with a higher rate of cardiac arrest in this study. Previous studies also demonstrated that poor nutrition and lower albumin levels were correlated with a higher rate of cardiac arrest and mortality [12]. Its decrease can be regarded as an acute-phase oxidative stress or inflammatory marker for infection [13]. A previous study did not show a significant benefit of using albumin for the resuscitation in sepsis condition [26]. Although in this study it was only barely significant due to the small sample size, the use of albumin in resuscitation of emergent hemodialysis patients may be further investigated.

The strength of this study was the timely and early risk stratification of emergent hemodialysis patients immediately after the patient entered the emergency department. Previous studies utilized early warning systems such as NEWS and MEWS with a red flag system and reaction teams to reduce IHCA. These studies required repeated measurement of vital signs, and dynamic change often occurred late to time peri-arrest [15]. Half of the patients were still in the low MEWS group 8 h prior to cardiac arrest, suggesting that monitoring the MEWS alone is not enough to predict cardiac arrest [27].

Prediction of cardiac arrest was only acceptable in NEWS and MEWS, with AUC for cardiac arrest reported to be 0.72, 0.69, respectively [14,16]. Studies have shown that increasing the biochemical parameters would increase the AUC for prediction events [28].

This study took advantage of the emergency triage system and incorporated biochemical parameters, all of which were easily available in the ED. Risk allocation and resource management can start immediately after the patient arrives at the hospital.

There have been no comparable studies on risk stratification in emergent hemodialysis patients. Hospitals in the United States provided emergency hemodialysis to patients and immigrants outside insurance coverage and had higher adverse events and mortality than regular hemodialysis [7]. The risk score for emergent cases was developed and validated in this study. The presentation of the scoring system followed the transparent reporting of a multivariable prediction model for individual prognosis or diagnosis method, along with internal validation, strengthened its application [29].

A risk-stratification strategy can be applied in the emergency department using our scoring system. When the data of the four factors were obtained, an early alarming system can be subsequently activated for high-risk patients. Patients with an initial high score would trigger alarm. A collaborative team that consists of emergency physicians, nephrologists, and intensive care specialists should provide more intensive care, including advanced monitoring, prompt specialist consultation, earlier intensive unit admission, earlier dialysis, shifting the dialysis mode to continuous hemodialysis, decreasing the volume of filtration, or adjustment of dialysates. An early alarming system would facilitate team coordination and patient care. These modifications provide future investigation interests, which could utilize this scoring system as an early alarming trigger.

### Limitations

Our study had several limitations. First, although this was a retrospective cohort study, the enrolled patients were from a single tertiary medical center with a small sample size. Further research and multi-center participants are needed to examine the prediction model in the future. The heterogenicity of enrollees may render a subgroup analysis. Second, there was a prevalence-incidence bias belonging to a type of selection bias, the Neyman bias, because we recruited patients that required emergency hemodialysis and experienced IHCA. Finally, this study was conducted in an Asian population with a relatively high prevalence of ESRD. The generalizability of our study results should be validated in other populations.

## 5. Conclusions

We found two triage variables (oxygen saturation < 85%, and MAP < 80 mmHg), and two biochemical variables (K > 5.5 mmol/L, and pH < 7.35) significantly contributing to the prediction score model for 72-h IHCA risk in an emergent hemodialysis patient cohort (AUC = 0.78, *p* < 0.001). The relative risk of cardiac arrest was 66.7% when the model score was 5, but 47.2% in the primary and 41.7% in the validation database when the model score was ≥3. This was the first study to demonstrate a clinically useful prediction score for IHCA in an emergency hemodialysis setting. Further studies are required to validate this scoring system and to utilize it as daily ED practice. Investigation on resuscitation and hemodialysis settings and details are required to further identify critical points to prevent this detrimental outcome.

## Figures and Tables

**Table 1:** The characteristics of the study subjects and underlying comorbidity domain for the incidence of in-hospital cardiac arrest (IHCA) with COX model.

IHCA within 3 Days	No (*n* = 146)		Yes (*n* = 44)	*p* Value	Incidence	Coefficient β	Univariate HR (95% CI)	Full Model HR (95% CI)	Final Model HR (95% CI)
Sex						−0.066			
Female	58 (39.7%)		18 (40.9%)	0.888	23.7%		1.0		
Male	88 (60.3%)		26 (59.1%)		22.8%		0.937 (0.513–1.708)		
Age (mean ± SD; year)	65.82 ± 13.68		67.14 ± 15.54	0.587		0.007	1.007 (0.985–1.029)		
Hypertension	No	43 (29.5%)	20 (45.5%)	0.048 *	31.7%		1.0	1.0	1.0
	Yes	103 (70.5%)	24 (54.5%)		18.9%	−0.639	0.528 (0.291–0.955) *	0.563 (0.310–1.022)	0.528 (0.291–0.955) *
DM	No	59 (40.4%)	16 (36.4%)	0.630	21.3%		1.0		
	Yes	87 (59.6%)	28 (63.6%)		24.3%	0.114	1.121 (0.606–2.071)		
CAD	No	91 (62.3%)	28 (63.6%)	0.875	23.5%		1.0		
	Yes	55 (37.7%)	16 (36.4%)		22.5%	−0.084	0.919 (0.497–1.699)		
CVA	No	119 (81.5%)	37 (84.1%)	0.695	23.7%		1.0		
	Yes	27 (18.5%)	7 (15.9%)		20.6%	−0.199	0.820 (0.365–1.838)		
CKD	Stage 1 or 2	26 (17.8%)	9 (20.5%)	0.791	25.7%		1.0		
	Stage 3 or 4	10 (6.8%)	4 (9.1%)		28.6%	0.044	1.045 (0.322–3.392)		
	Stage 5	110 (75.3%)	31 (70.5%)		22.0%	−0.274	0.760 (0.362–1.597)		
Malignancy	No	126 (86.3%)	40 (90.9%)	0.420	24.1%		1.0		
	Yes	20 (13.7%)	4 (9.1%)		16.7%	−0.341	0.711 (0.254–1.987)		
Heart failure	No	99 (67.8%)	36 (81.8%)	0.072	26.7%		1.0	1.0	
	Yes	47 (32.2%)	8 (18.2%)		14.5%	−0.709	0.492 (0.229–1.059)	0.530 (0.245–1.147)	

CKD stage 1 or 2 refers to GFR ≥ 60 mL/min/1.73 m^2^. CKD stage 3 or 4 refers to GFR 15–59 mL/min/1.73 m^2^. CKD stage 5 refers to GFR< 15 mL/min/1.73 m^2^. Statistical analysis is presented as * *p* < 0.05. Abbreviations were given as HR: hazard ratio; CI: confidence interval; SD: standard deviation; DM: diabetes mellitus; CAD: coronary artery disease; CVA: cerebral vascular accident; CKD: chronic kidney disease.

**Table 2:** The characteristics of the triage domain for the in-hospital cardiac arrest (IHCA) risk with COX model.

IHCA within 3 Days	No (*n* = 146)	Yes (*n* = 44)	*p* Value	Coefficient β	Univariate HR (95% CI)	Full Model HR (95% CI)	Final Model HR (95% CI)
Body temperature (°C)	36.56 ± 1.10	36.77 ± 1.37	0.364	0.110	1.117 (0.860–1.450)		
Pulse rate (beat/min)	91.85 ± 26.04	85.30 ± 31.77	0.172	−0.007	0.993 (0.982–1.004)		
Respiratory rate (cycle/min)	24.26 ± 7.65	24.00 ± 11.20	0.861	−0.005	0.995 (0.958–1.033)		
Systolic blood pressure (mmHg)	144.44 ± 43.82	120.33 ± 42.86	0.002 **	−0.011	0.989 (0.983–0.996) **	0.993 (0.980–1.007)	0.991 (0.984–0.999) *
Diastolic blood pressure (mmHg)	79.52 ± 28.02	66.90 ± 26.95	0.011 *	−0.015	0.985 (0.974–0.996) **	0.999 (0.977–1.021)	
Mean arterial pressure (mmHg)	101.16 ± 31.86	84.71 ± 30.96	0.004 **	−0.014	0.986 (0.977–0.995) **		
Oxygen saturation (%)	90.47 ± 12.28	85.83 ± 12.57	0.039 *	−0.017	0.983 (0.967–1.000) *	0.993 (0.973–1.012)	
Finger sugar (mg/dL)	184.27 ± 132.57	173.35 ± 83.50	0.735	−0.001	0.999 (0.995–1.003)		
Glasgow Coma Scale (GCS)	12.74 ± 3.85	11.91 ± 4.01	0.216	−0.049	0.952 (0.889–1.021)		
Eye opening (E)	3.58 ± 0.95	3.43 ± 1.04	0.370	−0.173	0.841 (0.639–1.107)		
Verbal response (V)	4.12 ± 1.55	3.60 ± 1.72	0.067	−0.147	0.863 (0.730–1.022)	0.927 (0.754–1.139)	
Motor response (M)	5.24 ± 1.54	4.95 ± 1.77	0.300	−0.095	0.909 (0.769–1.074)		

Quantitative data are presented as the mean ± standard deviation (SD). Hazard ratio (HR) was calculated as the number (95% confidence interval, CI). Statistical analysis is presented as * *p* < 0.05 and ** *p* < 0.01.

**Table 3:** The characteristics of the initial biochemistry results domain for the in-hospital cardiac arrest (IHCA) risk with COX model.

IHCA within 3 Days	No (*n* = 146)	Yes (*n* = 44)	*p* Value	Coefficient β	Univariate HR (95% CI)	Full Model HR (95% CI)	Final Model HR (95% CI)
WBC (10^3^/μL)	11.92 ± 5.65	14.08 ± 6.33	0.033 *	0.055	1.056 (1.009–1.105) *	1.044 (0.983–1.108)	1.064 (1.009–1.122) *
Hb (g/dL)	9.80 ± 2.50	11.04 ± 3.36	0.028 *	0.157	1.170 (1.050–1.304) **	1.064 (0.958–1.183)	
Plt (10^9^/L)	213.87 ± 90.48	185.74 ± 80.06	0.068	−0.003	0.997 (0.993–1.000)	0.995 (0.992–0.999) *	0.995 (0.991–0.999) **
PT-INR	1.19 ± 0.63	1.36 ± 0.84	0.190	0.165	1.179 (0.874–1.592)		
aPTT (sec)	39.36 ± 27.21	42.58 ± 33.56	0.563	0.004	1.004 (0.994–1.013)		
BUN (mg/dL)	101.75 ± 47.60	117.72 ± 77.70	0.416	0.005	1.005 (0.996–1.013)		
Cr (mg/dL)	8.33 ± 4.61	8.26 ± 5.47	0.947	−0.010	0.990 (0.916–1.069)		
eGFR (ml/min/1.73 m^2^)	9.01 ± 9.49	10.47 ± 15.23	0.520	0.018	1.019 (0.988–1.050)		
Lactate (mmol/L)	4.70 ± 5.21	5.93 ± 4.03	0.244	0.038	1.038 (0.980–1.100)		
Arterial gas							
pH	7.32 ± 0.16	7.23 ± 0.17	0.001 **	−2.527	0.080 (0.016–0.388) **	0.346(0.049–2.432)	
Base deficit (mmol/L)	−6.46 ± 7.54	−10.67 ± 7.21	0.002 **	−0.057	0.945 (0.912–0.979) **		
PCO_2_ (mmHg)	37.73 ± 17.51	39.45 ± 20.00	0.587	0.003	1.003 (0.988–1.019)		
PO_2_ (mmHg)	115.69 ± 95.49	93.00 ± 93.91	0.171	−0.003	0.997 (0.993–1.001)		
Na (mmol/L)	135.77 ± 6.54	139.09 ± 7.78	0.005 **	0.076	1.079 (1.035–1.126) ***	1.061 (1.014–1.110) **	1.069 (1.024–1.115) **
K (mmol/L)	4.83 ± 1.34	5.87 ± 1.83	0.001 **	0.280	1.323 (1.127–1.552) **	1.235 (1.037–1.470) *	1.296 (1.110–1.526) **
Ca (mg/dL)	8.78 ± 1.34	8.51 ± 1.96	0.521	−0.076	0.926 (0.706–1.215)		
Alb (g/dL)	3.85 ± 0.82	3.02 ± 1.09	0.044 *	−0.672	0.511 (0.246–1.059)		
Mg (mg/dL)	2.66 ± 0.82	2.82 ± 1.12	0.651	0.173	1.189 (0.637–2.217)		
TnT (ng/L)	0.40 ± 1.08	0.57 ± 1.59	0.482	0.093	1.097 (0.876–1.374)		
ALT (U/L)	51.49 ± 201.90	52.88 ± 102.98	0.966	0.000	1.000 (0.999–1.002)		

Quantitative data are presented as the mean ±standard deviation (SD). Hazard ratio (HR) was calculated as the number (95% confidence interval, CI). Abbreviations were given as WBC: white blood cell; Hb: hemoglobin; Plt: platelet; PT-INR: prothrombin time-international normalized ratio; aPTT, activated partial thromboplastin time; BUN, blood urea nitrogen; Cr, creatinine; eGFR, estimated glomerular filtration rate; Na, sodium; K, potassium; Ca, calcium; Alb, albumin; Mg, magnesium; TnT, troponin T; ALT, alanine aminotransferase. Statistical analysis is presented as * *p* < 0.05, ** *p* < 0.01, and *** *p* < 0.001.

**Table 4:** Establishment of the scoring system after rounding and dividing β coefficients from the final model from significant variables in each domain.

IHCA within 3 Days		No	Yes	*p* Value	Incidence	Univariate HR (95% CI)	Final Model HR (95% CI)	Coefficient β	Score
pH < 7.35	No	72 (53.7%)	11 (25%)	0.001 **	13.3%	1.0	1.0	0.686	1
	Yes	62 (46.3%)	33 (75%)		34.7%	2.768 (1.399–5.478) **	1.985 (0.968–4.073)		
K > 5.5 mmol/L	No	111 (77.6%)	20 (45.5%)	<0.001 ***	15.3%	1.0	1.0	0.521	1
	Yes	32 (22.4%)	24 (54.5%)		42.9%	2.943 (1.626–5.328) ***	1.683 (0.860–3.293)		
MAP < 80 mmHg	No	113 (81.3%)	21 (50.0%)	<0.001 ***	15.7%	1.0	1.0	0.872	2
	Yes	26 (18.7%)	21 (50.0%)		44.7%	3.475 (1.896–6.369) ***	2.392 (1.240–4.617) **		
Oxygen saturation < 85%	No	97 (75.8%)	22 (55.0%)	0.012	18.5%	1.0	1.0	0.515	1
	Yes	31 (24.2%)	18 (45.0%)		36.7%	2.182 (1.170–4.070) *	1.674 (0.866–3.238)		

Categorical variables are presented as numbers (percentages). Hazard ratio (HR) was presented as number (95% confidence interval [CI]). The cutoff value was determined to have the greatest area under the curve for predicting IHCA. The score was established based on the relative β coefficient ratio. Abbreviations were given as K: potassium; MAP: mean arterial pressure. Statistical analysis is presented as * *p* < 0.05, ** *p* < 0.01, and *** *p* < 0.001.

**Table 5:** The prediction score system and validation for the incidence of in-hospital cardiac arrest (IHCA).

IHCA within 3 Days	No	Yes	*p* Value	Incidence
Original model			<0.001	
Score = 0	36 (31.9%)	1 (2.6%)		2.7%
Score = 1	35 (31.0%)	5 (13.2%)		12.5%
Score = 2	23 (20.4%)	15 (39.5%)		39.5%
Score = 3	13 (11.5%)	9 (23.7%)		40.9%
Score = 4	4 (3.5%)	4 (10.5%)		50.0%
Score = 5	2 (1.8%)	4 (10.5%)		66.7%
Low-risk: Score < 3	94 (83.2%)	21 (55.3%)	<0.001	18.3%
High-risk: Score ≥ 3	19 (16.8%)	17 (44.7%)		47.2%
Validation model			0.042	
Score = 0	8 (17.0%)	0 (0.0%)		0.0%
Score = 1	20 (42.6%)	2 (25.0%)		9.1%
Score = 2	12 (25.5%)	1 (12.5%)		7.7%
Score = 3	4 (8.5%)	4 (50.0%)		50.0%
Score = 4	2 (4.3%)	1 (12.5%)		33.3%
Score = 5	1 (2.1%)	0 (0.0%)		0.0%
Low-risk: Score < 3	40 (85.1%)	3 (37.5%)	0.003	7.0%
High-risk: Score ≥ 3	7 (14.9%)	5 (62.5%)		41.7%

Categorical variables were presented as numbers (percentages). The scoring system was constructed from the original patient population between 2015 and 2018 and validated in the 2019 population. A cutoff value of 3 was determined to have the greatest differentiation. A score of 3 or above was regarded as a high-risk group, whereas a score below 3 was regarded as a low-risk group.

## Data Availability

Data can be made available on request from established research groups with an appropriate data-sharing agreement. Please contact the corresponding author for data sharing.

## Supplementary Materials

The following are available online at https://www.mdpi.com/article/10.3390/jcm10153241/s1, Table S1: The receiver-operating characteristic (ROC) curves for the incidence of IHCA between significant variables in each domain and predicting and validation models by comparing the area under curve (AUC) following by *p*-value, sensitivity, and specificity; Table S2: Sensitivity analysis of major factors between the primary (2015–2018) and validation (2019) cohorts; Table S3: Sensitivity analysis of major factors between the missing-data and complete-data groups.

## References

[1] Charytan D.M., Foley R., McCullough P.A., Rogers J.D., Zimetbaum P., Herzog C.A., Tumlin J.A. (2016). Arrhythmia and Sudden Death in Hemodialysis Patients: Protocol and Baseline Characteristics of the Monitoring in Dialysis Study. Clin. J. Am. Soc. Nephrol..

[2] Makar M.S., Pun P.H. (2017). Sudden Cardiac Death among Hemodialysis Patients. Am. J. Kidney Dis..

[3] Karnik J.A., Young B.S., Lew N.L., Herget M., Dubinsky C., Lazarus J.M., Chertow G.M. (2001). Cardiac arrest and sudden death in dialysis units. Kidney Int..

[4] Pun P.H., Lehrich R.W., Honeycutt E.F., Herzog C.A., Middleton J.P. (2011). Modifiable risk factors associated with sudden cardiac arrest within hemodialysis clinics. Kidney Int..

[5] Starks M.A., Wu J., Peterson E.D., Stafford J.A., Matsouaka R.A., Boulware E., Svetkey L.P., Chan P.S., Pun P.H. (2020). American Heart Association’s Get with the Guidelines-Resuscitation Investigators. In-Hospital Cardiac Arrest Resuscitation Practices and Outcomes in Maintenance Dialysis Patients. Clin. J. Am. Soc. Nephrol..

[6] Pun P.H., Lehrich R.W., Smith S.R., Middleton J.P. (2007). Predictors of Survival after Cardiac Arrest in Outpatient Hemodialysis Clinics. Clin. J. Am. Soc. Nephrol..

[7] Nguyen O.K., Vazquez M.A., Charles L., Berger J.R., Quiñones H., Fuquay R., Sanders J.M., Kapinos K.A., Halm E.A., Makam A.N. (2019). Association of Scheduled vs. Emergency-Only Dialysis with Health Outcomes and Costs in Undocumented Immigrants With End-stage Renal Disease. JAMA Intern. Med..

[8] Raghavan R. (2012). When Access to Chronic Dialysis is limited: One Center’s Approach to Emergent Hemodialysis. Semin. Dial..

[9] Lin Y.-C., Hsu H.-K., Lai T.-S., Chiang W.-C., Lin S.-L., Chen Y.-M., Chen C.-C., Chu T.-S. (2019). Emergency department utilization and resuscitation rate among patients receiving maintenance hemodialysis. J. Formos. Med. Assoc..

[10] Di Lullo L., Rivera R., Barbera V., Bellasi A., Cozzolino M., Russo D., De Pascalis A., Banerjee D., Floccari F., Ronco C. (2016). Sudden cardiac death and chronic kidney disease: From pathophysiology to treatment strategies. Int. J. Cardiol..

[11] Foley R.N., Parfrey P.S., Harnett J.D., Kent G.M., Murray D.C., Barre P.E. (1996). Hypoalbuminemia, cardiac morbidity, and mortality in end-stage renal disease. J. Am. Soc. Nephrol..

[12] Fung F., Sherrard D.J., Gillen D.L., Wong C., Kestenbaum B., Seliger S., Ball A., Stehman-Breen C. (2002). Increased risk for cardiovascular mortality among malnourished end-stage renal disease patients. Am. J. Kidney Dis..

[13] Alves F.C., Sun J., Qureshi A.R., Dai L., Snaedal S., Barany P., Heimbürger O., Lindholm B., Stenvinkel P. (2018). The higher mortality associated with low serum albumin is dependent on systemic inflammation in end-stage kidney disease. PLoS ONE.

[14] Smith G.B., Prytherch D., Meredith P., Schmidt P.E., Featherstone P.I. (2013). The ability of the National Early Warning Score (NEWS) to discriminate patients at risk of early cardiac arrest, unanticipated intensive care unit admission, and death. Resuscitation.

[15] Wang A.-Y., Fang C.-C., Chen S.-C., Tsai S.-H., Kao W.-F. (2016). Periarrest Modified Early Warning Score (MEWS) predicts the outcome of in-hospital cardiac arrest. J. Formos. Med. Assoc..

[16] Green M., Lander H., Snyder A., Hudson P., Churpek M., Edelson D. (2018). Comparison of the Between the Flags calling criteria to the MEWS, NEWS and the electronic Cardiac Arrest Risk Triage (eCART) score for the identification of deteriorating ward patients. Resuscitation.

[17] Lin C.-H., Tu Y.-F., Chiang W.-C., Wu S.-Y., Chang Y.-H., Chi C.-H. (2013). Electrolyte abnormalities and laboratory findings in patients with out-of-hospital cardiac arrest who have kidney disease. Am. J. Emerg. Med..

[18] Wu C., Hannan E.L., Walford G., Ambrose J.A., Holmes D.R., King S.B., Clark L.T., Katz S., Sharma S.K., Jones R.H. (2006). A Risk Score to Predict In-Hospital Mortality for Percutaneous Coronary Interventions. J. Am. Coll. Cardiol..

[19] Tsai C.-H., Tsai J.-L., Wang J.-Y. (2019). Feasibility of using Microsoft Excel to draw forest plots. Taiwan J. Public Health.

[20] Xu J.-Y., Ma S.-Q., Pan C., He H.-L., Cai S.-X., Hu S.-L., Liu A.-R., Liu L., Huang Y.-Z., Guo F.-M. (2015). A high mean arterial pressure target is associated with improved microcirculation in septic shock patients with previous hypertension: A prospective open label study. Crit. Care.

[21] Kim Y., Yun D., Kwon S., Jin K., Han S., Kim D.K., Oh K.-H., Joo K.W., Kim Y.S., Kim S. (2021). Target value of mean arterial pressure in patients undergoing continuous renal replacement therapy due to acute kidney injury. BMC Nephrol..

[22] McMahon G., Mendu M.L., Gibbons F.K., Christopher K.B. (2012). Association between hyperkalemia at critical care initiation and mortality. Intensiv. Care Med..

[23] Montford J.R., Linas S. (2017). How Dangerous Is Hyperkalemia?. J. Am. Soc. Nephrol..

[24] Kraut J.A., Madias N.E. (2016). Metabolic Acidosis of CKD: An Update. Am. J. Kidney Dis..

[25] Fujii T., Udy A., Licari E., Romero L., Bellomo R. (2019). Sodium bicarbonate therapy for critically ill patients with metabolic acidosis: A scoping and a systematic review. J. Crit. Care.

[26] Jiang L., Jiang S., Zhang M., Zheng Z., Ma Y. (2014). Albumin versus Other Fluids for Fluid Resuscitation in Patients with Sepsis: A Meta-Analysis. PLoS ONE.

[27] Kim W.Y., Shin Y.J., Lee J.M., Huh J.W., Koh Y., Lim C.-M., Hong S.B. (2015). Modified Early Warning Score Changes Prior to Cardiac Arrest in General Wards. PLoS ONE.

[28] Perera Y.S., Ranasinghe P., Adikari A.M., Welivita W.D., Perera W.M., Wijesundara W.M., Karunanayake S.A., Constantine G.R. (2011). The value of the Modified Early Warning Score and biochemical parameters as predictors of patient outcome in acute medical admissions a prospective study. Acute. Med..

[29] Collins G.S., Reitsma J.B., Altman D.G., Moons K.G.M. (2015). Transparent reporting of a multivariable prediction model for individual prognosis or diagnosis (TRIPOD): The TRIPOD Statement. BMC Med..

