# Supplementary material for: An Early Predictive Scoring Model for In-Hospital Cardiac Arrest of Emergent Hemodialysis Patients
Source: J Clin Med. 2021 Jul 22;10(15):3241. doi: 10.3390/jcm10153241 (PMC8347203; doi:10.3390/jcm10153241)
Supplement: Supplementary file 1 [file jcm-10-03241-s001.zip › jcm-1264076-supplementary.pdf]

Table S1. The receiver-operating characteristic (ROC) curves for the incidence of IHCA between significant variables in each domain and predicting and validation models by comparing the area under curve (AUC) following by *p*-value, sensitivity, and specificity.

IHCA within 3 Days	AUC	*p*-Value	Sensitivity	Specificity
pH < 7.35	0.644	0.004	0.507	0.727
K > 5.5 mmol/L	0.661	0.001	0.545	0.775
MAP < 80 mmHg	0.656	0.002	0.801	0.500
Saturation < 85%	0.604	0.048	0.789	0.425
Model of above 4 parameters	0.780	<0.001		
2019_validation model	0.753	0.023		

Selected parameters with greater area under the curve (AUC). Construction of the full model and validation in the 2019 database. Abbreviations were given as K: potassium; MAP: mean arterial pressure.

Table S2. Sensitivity analysis of major factors between the primary (2015–2018) and validation (2019) cohorts.

	Primary Database (*n* = 190)	Validation Database (*n* = 67)	*p* Value
pH < 7.35	*n* = 178	*n* = 64	
No	83 (46.6%)	26 (40.6%)	0.408
Yes	95 (53.4%)	38 (59.4%)	
K > 5.5 mmol/L	*n* = 187	*n* = 67	
No	131 (70.1%)	44 (65.7%)	0.506
Yes	56 (29.9%)	23 (34.3%)	
MAP < 80 mmHg	*n* = 181	*n* = 66	
No	134 (74.0%)	47 (71.2%)	0.658
Yes	47 (26.0%)	19 (28.8%)	
Oxygen saturation < 85%	*n* = 168	*n* = 57	
No	119 (70.8%)	45 (78.9%)	0.234
Yes	49 (29.2%)	12 (21.1%)	
Prediction score system	*n* = 151	*n* = 55	
Low-risk: Score < 3	115 (76.2%)	43 (78.2%)	0.751
High-risk: Score ≧ 3	36 (23.8%)	12 (21.8%)	
Complete model	*n* = 190	*n* = 67	
No (missing factor)	39 (20.5%)	12 (17.9%)	0.644
Yes (four factors)	151 (79.5%)	55 (82.1%)	

Abbreviations were given as K: potassium; MAP: mean arterial pressure.

Table S3. Sensitivity analysis of major factors between the missing-data and complete-data groups.

	**Missing-Data Group** (***n* = 51**)	**Complete-Data Group** (***n* = 206**)	***p* Value**
pH < 7.35	*n* = 36	*n* = 206	
No	12 (33.3%)	97 (47.1%)	0.126
Yes	24 (66.7%)	109 (52.9%)	
K > 5.5 mmol/L	*n* = 48	*n* = 206	
No	28 (58.3%)	147 (71.4%)	0.079
Yes	20 (41.7%)	59 (28.6%)	
MAP < 80 mmHg	*n* = 41	*n* = 206	
No	29 (70.7%)	152 (73.8%)	0.686
Yes	12 (29.3%)	54 (28.2%)	
Oxygen saturation < 85%	*n* = 19	*n* = 206	
No	15 (78.9%)	149 (72.3%)	0.535
Yes	4 (21.1%)	57 (27.7%)	
Prediction score system	*n* = 51	*n* = 206	
Low-risk: Score < 3	Not applicable	158 (76.7%)	
High-risk: Score ≥ 3	Not applicable	48 (23.3%)	

Abbreviations were given as K: potassium; MAP: mean arterial pressure.
